# Mechanical-Chemical Coupling Effects on an Environmental Barrier Coating System under High-Temperature Water Vapour Conditions

**DOI:** 10.3390/ma14195907

**Published:** 2021-10-08

**Authors:** Dingjun Li, Fan Sun, Cheng Ye, Peng Jiang, Jianpu Zhang, Yiwen Chen, Xiaohu Yuan

**Affiliations:** 1 State Key Laboratory for Strength and Vibration of Mechanical Structures, School of Aerospace Engineering, Xi’an Jiaotong University, Xi’an 710049, China; lidingjun@dongfang.com (D.L.); sf2038945624@163.com (F.S.); dmfuture@163.com (C.Y.); 2 State Key Laboratory of Long-Life High Temperature Materials, Dongfang Steam Turbine Co., Ltd., Deyang 618000, China; zhang695975990@126.com (J.Z.); chenyiwenac@163.com (Y.C.); yuanxiaohu@dongfang.com (X.Y.)

**Keywords:** environmental barrier coating, high-temperature water vapour, normalised chemical potential, normalised chemical reaction degree

## Abstract

The degradation mechanisms for environmental barrier coatings (EBCs) under high-temperature water vapour conditions are vital for the service of aero-engine blades. This study proposes a theoretical model of high-temperature water vapour corrosion coupled with deformation, mass diffusion and chemical reaction based on the continuum thermodynamics and the actual water vapour corrosion mechanisms of an EBC system. The theoretical model is suitable for solving the stress and strain fields, water vapour concentration distribution and coating corrosion degree of an EBC system during the water vapour corrosion process. The results show that the thickness of the corrosion zone on the top of the EBC system depended on water vapour diffusion, which had the greatest influence on the corrosion process. The top corroded area of the rare-earth silicate EBC system was significantly evident, and there was a clear dividing line between the un-corroded and corroded regions.

## 1. Introduction

To satisfy the requirements of a high thrust-to-weight ratio and low pollution for next-generation aero-engines, continuous silicon carbide fibre-reinforced silicon carbide ceramic matrix composites (SiC/SiC CMCs) have been gradually applied to replace the conventional nickel-based superalloy to enhance the turbine inlet temperature [1,2,3,4]. Although the high-temperature thermomechanical properties of SiC/SiC CMC are excellent, SiC/SiC CMC inevitably experiences extremely serious corrosion under the coupled action of oxygen, high-pressure water vapour and molten salt impurities when exposed to high-temperature conditions [5,6,7]. To isolate SiC/SiC CMC from the high-temperature environment inside the engine, the rare-earth silicate environmental barrier coating (EBC) improves corrosion resistance [8,9]. However, there still exists a certain degree of steam corrosion volatilisation in aero-engines, which results in a thinning and porous surface layer of EBC, leading to its performance degradation [10,11,12].

The performance degradation mechanisms for EBC under high-temperature water vapour conditions are important for the safe service of SiC/SiC CMC. To characterise the corrosion of rare-earth silicate in high-temperature water vapour, a series of simplified experimental devices for simulating the engine gas environment have been established, and the mass loss rate of rare-earth silicate materials has been investigated [13,14,15,16]. However, these experimental devices are unable to simulate the actual high-temperature and high-pressure gas in the laboratory. In addition, it is difficult to capture the real-time performance variation of the EBC system, i.e., gas impurities and sample quality, during the experiment. A few researchers have established theoretical models to describe the corrosion process of the EBC system under high-temperature water vapour conditions. Opila and co-workers [17] proved that silicon matrix composites are severely corroded by water vapour at high-temperature conditions and established a model using the plate convection–diffusion theory to describe the linear corrosion kinetics of silicon-based ceramics under high-temperature and high-speed water vapour conditions, as well as the evolution mechanisms of the surface oxide layer thickness. Jacobson and Costa [18,19] obtained the linear corrosion kinetics of yttrium disilicate and monociliate based on a plate diffusion model. In contrast, they observed some errors between the theoretical prediction and experimental data because the effect of the porous structure on the corrosion process was not considered.

In this study, a theoretical model considering coupling deformation, mass diffusion and chemical reactions was developed to obtain the stress/strain field, water vapour concentration distribution and corrosion degree distribution of the EBC system from the initial state to the steam corrosion process. The dependence of the EBC on the time scale of the diffusion and reaction processes in steam corrosion is discussed in detail. The model proposed here may provide better guidance for the design of EBC systems.

## 2. Theoretical Framework of Continuum Thermodynamics

### 2.1. Laws of Thermodynamics and the Dissipation Function

A macroscopically homogeneous and isotropic open system consisting of chemically active media was considered. The continuous medium was assumed to accept the diffusion input and output of the external material and react with the external material simultaneously. Each element in the continuum was considered autonomous and uniform, and the entire system was regarded as an integral superposition of elements.

According to the Euler method, the internal energy, E, and kinetic energy, K, of the object are expressed as follows:

(1) $$E=\int_{B}\mathrm{edV}$$

(2) $$K=\int_{B}\rho v\cdot v\mathrm{dV}$$

where ρ, e and v represent the mass density, internal energy per unit volume and velocity per unit mass, respectively. B and V represent the object and volume element of the object, respectively.

The work performed by the internal and external forces on the object per unit time is expressed as follows:

(3) $$\dot{W}=\int_{B}\rho f\cdot v\mathrm{dV}+\int_{\partial B}t\cdot v\mathrm{dS}$$

where the dot over W represents the differentiation concerning time, and f and t represent the body force per unit mass and traction per unit area, respectively.

Similarly, the heat transferred to the system through the environment per unit time and the heat generated within the system are expressed as follows:

(4) $$\dot{Q}=\int_{B}\mathrm{rdV}-\int_{\partial B}q\cdot n\mathrm{dS}$$

where r represents the heat generated per unit volume inside the body, q represents the external heat flow vector of the object surface and n denotes the outward-drawn unit normal vector.

Furthermore, when a matter diffuses into or out of the body, the diffusing matter provides or consumes the energy of the thermodynamic system, which is expressed as:

(5) $$E_{\mathrm{diffusion}}=-\underset{\partial B}{\sum }\mu_{\alpha }j_{\alpha }^{m}\cdot n\mathrm{dS}$$

where μ represents the chemical potential of the diffusing matter, α indicates the type of diffusing matter and j denotes the diffusion flux vector, which is positive when it diffuses outward. The superscript m denotes that the diffusion flux vector is a matter flux vector.

Based on the first law of thermodynamics, the increase in the internal energy of the system is equal to the sum of the absorbed heat and the work performed on the system, which satisfies the following equation:

(6) $$\frac{d}{\mathrm{dt}}\int_{B}\left(e+\rho \frac{1}{2}v\cdot v\right)\mathrm{dV}=\int_{B}\left(\rho f\cdot v+r\right)\mathrm{dV}+\int_{\partial B}(t\cdot v-q\cdot n-\underset{\partial B}{\sum }\mu_{\alpha }j_{\alpha }^{m}\cdot n)\mathrm{dS}$$

According to the Reynolds transmission theorem and the Cauchy equation, t=σ, Equation (6) is adjusted as follows:

(7) $$\int_{B}\left[\sigma :\mathrm{grad}v-\mathrm{div}q+r-\mathrm{div}(\underset{\alpha }{\sum }\mu_{\alpha }j_{\alpha }^{m})-\dot{e}\right]\mathrm{dV}+\int_{B}\left[v\cdot (\mathrm{div}\sigma +\rho f-\rho \dot{v}\right]\mathrm{dV}=0$$

According to the principle of momentum conservation,

(8) $$\rho \dot{v}=\mathrm{div}\sigma +\rho f$$

Moreover, when chemical reactions and material diffusion exist, the mass conservation can be expressed as:

(9) $$\frac{d}{\mathrm{dt}}\int_{B}\dot{\bar{c_{\alpha }}}\mathrm{dV}=-\int_{\partial B}j_{\alpha }^{m}\cdot n\mathrm{dS}+\int_{B}{\dot{r}}_{\to \alpha }\mathrm{dV}$$

where $\bar{c}$ refers to the mass concentration of the diffusing substance, and the second integral term indicates the rate of molecular formation per unit volume. When the second term is greater than 0, a chemical reaction produces matter α; when it less than 0, a chemical reaction consumes matter α.

Given the quasi-static state of the object and the randomness of different regions, the integral form of Equation (9) is removed by using the divergence theorem, and the local mass balance equation in the object can be written as:

(10) $$\dot{\bar{c_{\alpha }}}=-\mathrm{div}\left(j_{\alpha }^{m}\right)+{\dot{r}}_{\to \alpha }$$

Equation (7) can be summarised by introducing Equation (8) and Equation (10):

(11) $$\dot{e}=\sigma :\mathrm{grad}v-\mathrm{div}q+r+\underset{\alpha }{\sum }\mu_{\alpha }\dot{\bar{c_{\alpha }}}-\underset{\alpha }{\sum }\mu_{\alpha }{\dot{r}}_{\to \alpha }-\underset{\alpha }{\sum }j_{\alpha }^{m}\cdot \mathrm{div}\left(\mu_{\alpha }\right)$$

Under the condition of small deformation, the velocity gradient is expressed by the strain rate.

(12) $$\dot{e}=\sigma :\dot{\varepsilon }-\nabla \cdot q+r+\underset{\alpha }{\sum }\mu_{\alpha }\dot{\bar{c_{\alpha }}}-\underset{\alpha }{\sum }\mu_{\alpha }{\dot{r}}_{\to \alpha }-\underset{\alpha }{\sum }j_{\alpha }^{m}\cdot \nabla \left(\mu_{\alpha }\right)$$

Equation (12) describes the energy conservation law for the thermodynamic process. However, to describe a non-equilibrium continuum thermodynamics system, the direction of the thermodynamic process was also considered. According to the second law of thermodynamics, the entropy growth rate of a system is not less than the entropy growth rate owing to the heat source and heat flow. Thus, the total entropy increase rate of a thermodynamic system satisfies the following inequality:

(13) $$\frac{\mathrm{dS}}{\mathrm{dt}}\geq \int_{B}\frac{r}{T}\mathrm{dV}-\int_{\partial B}\frac{q\cdot n}{T}\mathrm{dS}$$

where S is the entropy of the thermodynamic system, and $\frac{r}{T}$ and $\frac{q}{T}$ represent the entropy increase caused by internal heat generation and external heat flow and known as an entropy source and flow, respectively. The inequality sign in Equation (13) is replaced with the equal sign for a reversible process.

Equation (13) can be summarised using the divergence and Reynolds transport theorems:

(14) $$\int_{B}\left[\dot{s}+\mathrm{div}\frac{q}{T}-\frac{r}{T}\mathrm{dV}\right]\geq 0$$

where s is the entropy per unit mass of the object, and T is the absolute temperature.

The entropy mentioned above increase relation applies to any region of the object, and all partial forms are expressed as:

(15) $$\dot{s}+\mathrm{div}\frac{q}{T}-\frac{r}{T}\mathrm{dV}\geq 0$$

The following relation is obtained by introducing Equation (12) into Equation (15):

(16) $$\sigma :\dot{\varepsilon }+T\dot{s}-\dot{e}-q\cdot \mathrm{gradT}/T+(\underset{\alpha }{\sum }\mu_{\alpha }\dot{\bar{c_{\alpha }}}-\underset{\alpha }{\sum }\mu_{\alpha }{\dot{r}}_{\to \alpha }-\underset{\alpha }{\sum }j_{\alpha }^{m}\cdot \nabla \mu_{\alpha })\geq 0$$

Introducing the Helmholtz free energy per unit volume results in:

(17) ψ=e−Ts

The internal energy, e, of the system is eliminated, and the Clausius–Duhem inequality applicable to the problem is derived as follows:

(18) $$\Phi =\sigma :\dot{\varepsilon }-T\dot{s}-\dot{\psi }-q\cdot \mathrm{gradT}/T+\left(\underset{\alpha }{\sum }\mu_{\alpha }\dot{\bar{c_{\alpha }}}-\underset{\alpha }{\sum }\mu_{\alpha }{\dot{r}}_{\to \alpha }-\underset{\alpha }{\sum }j_{\alpha }^{m}\cdot \nabla \mu_{\alpha }\right)\geq 0$$

where Φ denotes the unit mass of the object in the dissipation function. The inequality is the most basic constraint of constitutive equations and provides a basis for the thermodynamic constitutive theory of the continuum dissipative process.

### 2.2. Thermodynamic Potential and Constitutive Equation

For a thermodynamic system with chemical reactions, the change in the concentration of matter is composed of the diffusion concentration of the matter and the influence of the chemical reaction. To simplify the equation, the physical quantity, c_α_, was employed to express the concentration of a substance diffused into the object from outside; hence, Equation (18) is simplified as follows:

(19) $$\Phi =\sigma :\dot{\varepsilon }-\dot{T}s-\dot{\psi }-q\cdot \mathrm{gradT}/T+\left(\underset{\alpha }{\sum }\mu_{\alpha }{\dot{c}}_{\alpha }-\underset{\alpha }{\sum }j_{\alpha }^{m}\cdot \nabla \mu_{\alpha }\right)\geq 0$$

Given the finite deformation of the object, the total strain is considered a simple linear summation composed of elastic, thermal, chemical and other inelastic strains. The total strain is expressed as follows:

(20) $$\varepsilon =\varepsilon^{e}+\varepsilon^{T}+\varepsilon^{c}+\varepsilon^{i}$$

where the chemical strain, $\varepsilon^{c},$ includes volumetric expansion caused by the absorption of diffusing substances and volumetric contraction or expansion caused by chemical reactions. 

The total strain, the concentration of the diffused substance, absolute temperature, degree of chemical reaction and other dissipative effects were considered the independent state variables of the thermodynamic objects in this study. Therefore, the free energy density is expressed as a scalar function of state variables:

(21) $$\Psi =\Psi \left(\varepsilon_{\mathrm{ij}},c_{\alpha },T,l_{\gamma },\chi \right)$$

where γ represents the serial number of a chemical reaction suitable for multiple chemical reaction processes occurring simultaneously. Equation (21) characterises the energy distribution caused by the total strain, absorption and chemical reaction of the diffusing material, temperature change and dissipation of internal variables except for the chemical processes.

Although the diffusion concentration of substances, $c_{\alpha },$ and the degree of chemical reaction, $l_{\gamma },$ possess a certain correlation, these correspond to the diffusion and chemical reaction of substances, respectively, which are considered two different chemical processes and cause variation in the structure and performance. Therefore, $c_{\alpha }$ and $l_{\gamma }$ were still used as two independent state variables to describe the energy contribution of the diffusion material entering the object and the subsequent chemical reaction between the diffusion material and the object.

Substituting Equation (21) into the dissipation inequality in Equation (17) leads to:

(22) $$\left(\sigma_{\mathrm{ij}}-\frac{\partial \Psi }{\partial \varepsilon_{\mathrm{ij}}}\right){\dot{\varepsilon }}_{\mathrm{ij}}+\underset{\alpha }{\sum }(\mu_{\alpha }-\frac{\partial \Psi }{\partial c_{\alpha }}){\dot{c}}_{\alpha }-\left(s+\frac{\partial \Psi }{\partial T}\right)\dot{T}+\underset{\gamma }{\sum }A_{\gamma }{\dot{l}}_{\gamma }+\underset{\gamma }{\sum }\Gamma_{n}\dot{\chi_{n}}-q\cdot \mathrm{gradT}/T-\underset{\alpha }{\sum }j_{\alpha }^{m}\cdot \nabla \mu_{\alpha }\geq 0$$

$$A_{\gamma }=-{\left(\frac{\partial \Psi }{\partial l_{\gamma }}\right)}_{\varepsilon_{\mathrm{ij},c_{\alpha },T,\chi_{n}}}$$

(23) $$\Gamma_{n}=-{\left(\frac{\partial \Psi }{\partial \chi_{n}}\right)}_{\varepsilon_{\mathrm{ij},c_{\alpha },T,l_{\gamma }}}$$

where $A_{\gamma }$ is defined as the chemical affinity of a chemical reaction, representing the generalised conjugation force of the degree of chemical reaction, $l_{\gamma }$, and expresses the change in free energy per unit degree of reaction. $\Gamma_{n}$ also corresponds to the generalised conjugate force of other dissipative effects, $\chi_{n}$, which represents the change in free energy per unit of dissipative effects.

Based on the assumption that stress, chemical potential and entropy are independent of the strain, concentration change and temperature change rates, the latter’s value should satisfy Equality (22) and the first three coefficients on the left side of Equation (22) are 0. The constitutive equation is expressed as follows:

(24) $$\sigma_{\mathrm{ij}}={\left(\frac{\partial \Psi }{\partial \varepsilon_{\mathrm{ij}}}\right)}_{c_{\alpha },T,l_{\gamma },\chi_{n}},\;\mu_{\alpha }={\left(\frac{\partial \Psi }{\partial c_{\alpha }}\right)}_{\varepsilon_{\mathrm{ij}},T,l_{\gamma },\chi_{n}},\;s=-{\left(\frac{\partial \Psi }{\partial T}\right)}_{\varepsilon_{\mathrm{ij}},c_{\alpha },l_{\gamma },\chi_{n}}$$

According to Equations (23) and (24), the corresponding Maxwell symmetry relationship is as follows:

$$\frac{\partial \sigma_{\mathrm{ij}}}{\partial \varepsilon_{\mathrm{kl}}}=\frac{\partial \sigma_{\mathrm{kl}}}{\partial \varepsilon_{\mathrm{ij}}},\;\frac{\partial \sigma_{\mathrm{ij}}}{\partial c_{\alpha }}=\frac{\partial \mu_{\alpha }}{\partial \varepsilon_{\mathrm{ij}}},\;\frac{\partial \sigma_{\mathrm{ij}}}{\partial T}=-\frac{\partial s}{\partial \varepsilon_{\mathrm{ij}}},\;\frac{\partial \sigma_{\mathrm{ij}}}{\partial l_{\gamma }}=-\frac{\partial A_{\gamma }}{\partial \varepsilon_{\mathrm{ij}}},\;\frac{\partial \sigma_{\mathrm{ij}}}{\partial \chi_{n}}=-\frac{\partial \Gamma_{n}}{\partial \varepsilon_{\mathrm{ij}}}$$

$$\frac{\partial \mu_{\alpha }}{\partial T}=-\frac{\partial s}{\partial c_{\alpha }},\;\frac{\partial \mu_{\alpha }}{\partial \chi_{n}}=-\frac{\partial \Gamma_{n}}{\partial c_{\alpha }}$$

$$\frac{\partial s}{\partial l_{\gamma }}=\frac{\partial A_{\gamma }}{\partial T},\;\frac{\partial s}{\partial \chi_{n}}=\frac{\partial \Gamma_{n}}{\partial T}$$

(25) $$\frac{\partial A_{\gamma }}{\partial c_{\alpha }}=-\frac{\partial \mu_{\alpha }}{\partial l_{\gamma }},\;\frac{\partial A_{\gamma }}{\partial \chi_{n}}=\frac{\partial \Gamma_{n}}{\partial l_{\gamma }}$$

The incremental form of the constitutive equation in the thermomechanical coupling system and the corresponding linear form are obtained as follows by comprehensively comparing Equations (22) and (23):

$${d\sigma }_{\mathrm{ij}}=C_{\mathrm{ijkl}}{d\varepsilon }_{\mathrm{kl}}+\underset{\alpha }{\sum }R_{\mathrm{ij}}^{\alpha }{\mathrm{dc}}_{\alpha }+\lambda_{\mathrm{ij}}\mathrm{dT}-C_{\mathrm{ijkl}}\underset{\gamma }{\sum }\beta_{\mathrm{kl}}^{\gamma }{\mathrm{dl}}_{\gamma }-\underset{n}{\sum }\zeta_{n}^{\sigma }{d\chi }_{n}$$

$${d\mu }_{\alpha }=R_{\mathrm{ij}}^{\alpha }{d\varepsilon }_{\mathrm{ij}}+N^{\alpha }{\mathrm{dc}}_{\alpha }+v^{\alpha }\mathrm{dT}-\underset{\gamma }{\sum }\zeta_{\alpha }^{\gamma }{\mathrm{dl}}_{\gamma }+\underset{n}{\sum }\zeta_{n}^{\mu }{d\chi }_{n}$$

$$\mathrm{ds}=\lambda_{\mathrm{ij}}{d\varepsilon }_{\mathrm{ij}}-\underset{\alpha }{\sum }v^{\alpha }{\mathrm{dc}}_{\alpha }+C\frac{\mathrm{dT}}{T}+\frac{1}{T}\underset{\gamma }{\sum }L_{\gamma }{\mathrm{dl}}_{\gamma }+\frac{1}{T}\underset{n}{\sum }\zeta_{n}^{T}{d\chi }_{n}$$

$${\mathrm{dA}}_{\gamma }=C_{\mathrm{ijkl}}\beta_{\mathrm{kl}}^{\gamma }{d\varepsilon }_{\mathrm{ij}}+\underset{\alpha }{\sum }\zeta_{\alpha }^{\gamma }{\mathrm{dc}}_{\alpha }+L_{\gamma }\frac{\mathrm{dT}}{T}+\alpha_{\gamma }{\mathrm{dl}}_{\gamma }+\underset{n}{\sum }\zeta_{n}^{A}{d\chi }_{n}$$

$${d\Gamma }_{n}=\zeta_{n}^{\sigma }{d\varepsilon }_{\mathrm{ij}}+\underset{\alpha }{\sum }\zeta_{n}^{\mu }{\mathrm{dc}}_{\alpha }+\zeta_{n}^{T}\frac{\mathrm{dT}}{T}+\underset{\gamma }{\sum }\zeta_{n}^{A}{\mathrm{dl}}_{\gamma }+\pi_{n}{d\chi }_{n}$$

$$\sigma_{\mathrm{ij}}-\sigma_{\mathrm{ij}}^{0}=C_{\mathrm{ijkl}}\varepsilon_{\mathrm{kl}}+\underset{\alpha }{\sum }R_{\mathrm{ij}}^{\alpha }(c_{\alpha }-c_{\alpha }{}^{0})-\lambda_{\mathrm{ij}}\left(T-T^{0}\right)-C_{\mathrm{ijkl}}\underset{\gamma }{\sum }\beta_{\mathrm{kl}}^{\gamma }(l_{\gamma }-l_{\gamma }{}^{0})-\underset{n}{\sum }\zeta_{n}^{\sigma }(\chi_{n}-\chi_{n}{}^{0})$$

$$\mu_{\alpha }-\mu_{\alpha }{}^{0}=R_{\mathrm{ij}}^{\alpha }\varepsilon_{\mathrm{ij}}+N^{\alpha }\left(c_{\alpha }-c_{\alpha }{}^{0}\right)+v^{\alpha }\left(T-T^{0}\right)-\underset{\gamma }{\sum }\zeta_{\alpha }^{\gamma }(l_{\gamma }-l_{\gamma }{}^{0})+\underset{n}{\sum }\zeta_{n}^{\mu }(\chi_{n}-\chi_{n}{}^{0})$$

$$s-s^{0}=\lambda_{\mathrm{ij}}\varepsilon_{\mathrm{ij}}-\underset{\alpha }{\sum }v^{\alpha }(c_{\alpha }-c_{\alpha }{}^{0})+C\frac{\left(T-T^{0}\right)}{T}+\frac{1}{T}\underset{\gamma }{\sum }L_{\gamma }(l_{\gamma }-l_{\gamma }{}^{0})+\frac{1}{T}\underset{n}{\sum }\zeta_{n}^{T}\left(\chi_{n}-\chi_{n}{}^{0}\right)$$

$$A_{\gamma }-A_{\gamma }{}^{0}=C_{\mathrm{ijkl}}\beta_{\mathrm{kl}}^{\gamma }\varepsilon_{\mathrm{ij}}+\underset{\alpha }{\sum }\zeta_{\alpha }^{\gamma }\left(c_{\alpha }-c_{\alpha }{}^{0}\right)+L_{\gamma }\frac{\left(T-T^{0}\right)}{T}+\alpha_{\gamma }\left(l_{\gamma }-l_{\gamma }{}^{0}\right)+\underset{n}{\sum }\zeta_{n}^{A}(\chi_{n}-\chi_{n}{}^{0})$$

(26) $$\Gamma_{n}-\Gamma_{n}{}^{0}=\zeta_{n}^{\sigma }\varepsilon_{\mathrm{ij}}+\underset{\alpha }{\sum }\zeta_{n}^{\mu }(c_{\alpha }-c_{\alpha }{}^{0})+\zeta_{n}^{T}\frac{\left(T-T^{0}\right)}{T}+\underset{\gamma }{\sum }\zeta_{n}^{A}\left(l_{\gamma }-l_{\gamma }{}^{0}\right)+\pi_{n}\left(\chi_{n}-\chi_{n}{}^{0}\right)$$

where the specific meaning of the corresponding material parameters is explained later when used in detail. Superscript 0 represents the initial state, assuming that the object has no initial strain.

## 3. Constitutive Equation of EBC during the Corrosion Process under High-temperature Water Vapour

Under the high-temperature water vapour corrosion process of EBC, only the isothermal process of gas is considered. When the thermal effect is ignored, and the diffusing substance molecules and the object receiving the diffusive substance are assumed to be compressible, the volumetric strain of EBC is equal to the sum of the expansion strain caused by the absorber and the contraction strain caused by the reaction, which is expressed as:

(27) $$\varepsilon_{\mathrm{kk}}=\Omega \left(c-c^{0}\right)+3\beta \left(l-l^{0}\right)$$

where Ω represents the molecular volume of the diffusion material, and β represents the volume shrinkage rate of the chemical reaction, which reflects the volume difference between the reactant and the product.

Under high-temperature water vapour conditions, the strain, concentration of the diffusing substance, temperature and degree of the chemical reaction should be considered as the internal state variables of EBC. Therefore, the free energy density is expressed as a state variable.

(28) $$\Psi =\Psi \left(\varepsilon_{\mathrm{kk}},e_{\mathrm{ij}},c,l\right)$$

As Equation (27) is a constraint equation, a Lagrangian multiplier, p, needs to be introduced into the free energy density function, and the new free energy density function is expressed as:

(29) $$W\left(\varepsilon_{\mathrm{kk}},e_{\mathrm{ij}},c,l,p\right)=\Psi \left(\varepsilon_{\mathrm{kk}},e_{\mathrm{ij}},c,l\right)+p\left[\varepsilon_{\mathrm{kk}}-\Omega \left(c-c^{0}\right)-3\beta \left(l-l^{0}\right)\right]$$

The dissipation inequality also needs to be rewritten as:

(30) $$\left(\sigma_{m}-p-\frac{\partial \Psi }{\partial \varepsilon_{\mathrm{kk}}}\right){\dot{\varepsilon }}_{\mathrm{kk}}+\left(s_{\mathrm{ij}}-\frac{\partial \Psi }{\partial e_{\mathrm{ij}}}\right){\dot{e}}_{\mathrm{ij}}+\left(\mu +p\Omega -\frac{\partial \Psi }{\partial c}\right)\dot{c}+\left(A+3\beta p\right)\dot{l}-j^{m}\cdot \nabla \mu \geq 0$$

where $\sigma_{m}$ denotes hydrostatic pressure, μ represents chemical potential and A is chemical affinity.

In Equation (30), when the first three coefficients become zero, the state equations are modified as follows:

(31) $$\sigma_{m}-p=\frac{\partial \Psi }{\partial \varepsilon_{\mathrm{kk}}};s_{\mathrm{ij}}=\frac{\partial \Psi }{\partial e_{\mathrm{ij}}};\mu +p\Omega =\frac{\partial \Psi }{\partial c}$$

According to Ottosen et al. [19], the EBC is used as a chemically active medium under isotropic conditions. After simplifying and removing irrelevant terms, the specific expression for the free energy density is as follows:

(32) $$\Psi =\Psi^{e}\left(\varepsilon ,e_{\mathrm{ij}}\right)+\Psi^{d}\left(c\right)+\Psi^{r}\left(l\right)+\Psi^{c}\left(\varepsilon ,c.l\right)$$

where G represents the shear modulus and Ψ^e^ indicates the elastic energy density, which can be expressed as:

(33) $$\Psi^{e}\left(\varepsilon ,e_{\mathrm{ij}}\right)=\sigma_{m}^{0}\varepsilon_{\mathrm{kk}}+s_{\mathrm{ij}}^{0}e_{\mathrm{ij}}+\frac{1}{2}{K\varepsilon }_{\mathrm{kk}}{}^{2}+G\left(e_{1}{}^{2}+e_{2}{}^{2}+e_{3}{}^{2}\right)$$

$\Psi^{d}\left(c\right)$ and $\Psi^{r}\left(l\right)$ represent the energy density related to the diffusion of matter and chemical reaction and can be expressed as Equations (34) and (35), respectively.

(34) $$\Psi^{d}\left(c\right)=\mu^{0}c+\frac{1}{2}N{\left(c-c^{0}\right)}^{2}$$

(35) $$\Psi^{r}\left(l\right)=-A^{0}l-\frac{1}{2}a{\left(l-l^{0}\right)}^{2}$$

$\Psi^{c}\left(\varepsilon ,c.l\right)$ represents the energy density related to chemical process coupling:

(36) $$\Psi^{c}\left(\varepsilon ,c,l\right)=-3K\beta \left(l-l^{0}\right)\varepsilon_{\mathrm{kk}}-\varsigma \left(c-c^{0}\right)\left(l-l^{0}\right)$$

As the relationship between molecular concentration and volume satisfies volume constraints in Equation (27), the coupling term of concentration and volume is not considered.

The isotropic linear constitutive equation can be obtained by substituting Equation (29) into the state equation represented by Equation (30):

$$\sigma_{\mathrm{kk}}-\sigma_{\mathrm{kk}}{}^{0}=3{K\varepsilon }_{\mathrm{kk}}-9K\beta \left(l-l^{0}\right)+3p$$

$$s_{\mathrm{ij}}-s_{\mathrm{ij}}{}^{0}=2{\mathrm{Ge}}_{\mathrm{ij}}$$

$$\mu -\mu^{0}=N\left(c-c^{0}\right)-\varsigma \left(l-l^{0}\right)-p\Omega$$

(37) $$A-A^{0}=3{K\beta \varepsilon }_{\mathrm{kk}}+\varsigma \left(c-c^{0}\right)+a\left(l-l^{0}\right)$$

Finally, the Lagrangian multiplier is eliminated by substituting the constraint Equation (27) into the first three equations of the input Equation (37):

(38) $$\sigma_{\mathrm{ij}}-\sigma_{\mathrm{ij}}{}^{0}={\tilde{\lambda }\varepsilon }_{\mathrm{kk}}\delta_{\mathrm{ij}}+2{G\varepsilon }_{\mathrm{ij}}-L\left(l-l^{0}\right)\delta_{\mathrm{ij}}-\frac{\mu -\mu^{0}}{\Omega }\delta_{\mathrm{ij}}$$

where $\tilde{\lambda }=\lambda +\frac{N}{\Omega^{2}},\;L=\frac{E\beta }{1-2v}+\frac{\varsigma }{\Omega }+\frac{3\beta N}{\Omega^{2}}$ and λ is a Lame constant.

**Figure 1:**
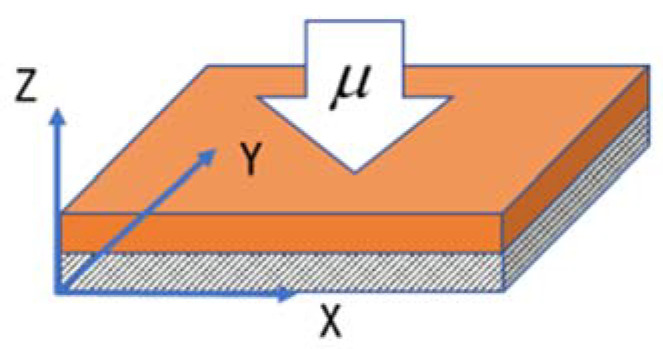
Schematic diagram of EBC with the substrate when corroded by high-temperature water vapour.

For the EBC with a substrate, water vapour diffused from the outer surface into the interior, and the deformation in the direction of the EBC plane was constrained by the substrate, as shown in Figure 1. The strain in both directions was 0, i.e., ε_x_ = ε_y_ = 0. When considering the isotropy, the stress in the two directions in the plane remained constant, that is, (z, t) = σ_y_(z, t). In the entire corrosion process, regardless of the geometric characteristics of the corrosion area after the rare-earth silicate reaction, a complete and uniform geometric morphology was still maintained. The out-of-plane normal stress of the EBC remained at 0. Under the condition of equal in-plane strain, the stress–strain constitutive relationship according to Equation (38) is expressed as follows:

(39) $$\varepsilon_{z}=\frac{\partial u_{z}}{\partial t}=\frac{1}{\tilde{\lambda }+2G}\left[\frac{\mu -\mu^{0}}{\Omega }+\mathrm{Ll}\right]\;\sigma_{x}=\sigma_{y}=\frac{-2G}{\tilde{\lambda }+2G}\left[\frac{\mu -\mu^{0}}{\Omega }+\mathrm{Ll}\right]$$

In thermodynamics, the driving force for water vapour diffusion in the EBC is mainly caused by the potential chemical gradient instead of the concentration gradient. For two macroscopic systems that can exchange particles and energy, the molecules always enter from a high to low chemical potential phase, which reduces the free energy of the system; consequently, the system reaches the final equilibrium state. Therefore, according to the potential chemical expression of Fick’s second law, the mass balance in the diffusion process can be expressed as follows:

(40) $$\frac{\partial c}{\partial t}=D^{*}\nabla^{2}\mu ,D=\kappa /{\eta \Omega }^{2},D^{*}=D{\left(1-\hat{l}\times 0.26\right)}^{-3/2}$$

where D represents the diffusion coefficient under the chemical potential, κ is the permeability of the coating, η is the viscosity of the diffusing substance and $D^{*}$ indicates the modified diffusion coefficient under the Bruggeman assumption, which considers the actual impact of the porous channel structure.

According to Equations (27), (39) and (40), the concentration term is eliminated, and the chemical potential is substituted to express the influence of the concentration term. The evolution equation of the chemical potential is:

(41) $$\frac{\partial \mu }{\partial t}=\Omega^{2}D^{*}\left(\tilde{\lambda }+2G\right)\frac{\partial^{2}\mu }{\partial z^{2}}+\left[3\beta \Omega \left(\tilde{\lambda }+2G\right)-\Omega L\right]\frac{\partial l}{\partial t}=D^{**}\frac{\partial^{2}\mu }{\partial z^{2}}+L^{*}\frac{\partial l}{\partial t}$$

Similarly, using Equation (27) to eliminate the concentration term in the chemical reaction kinetic equation dn/dt = kc results in:

(42) $$\frac{\partial l}{\partial t}=\mathrm{kc}=\left[\frac{k}{\Omega }\frac{\partial \mu_{z}}{\partial z}+\left(\vartheta -\frac{3\beta }{\Omega }\right)\mathrm{kl}\right]H\left(\bar{l}-l\right);\;H\left(x\right)=\{\begin{array}{c} 1,x>0\\ 0,x=0 \end{array}$$

where k is the rate constant of the reaction between rare-earth silicate and high-temperature water vapour, and $\bar{l}$ is the highest degree of the chemical reaction. When the chemical reaction is completed, the gradient of the chemical reaction degree is 0 and the chemical reaction does not proceed.

In conclusion, Equations (39), (41) and (42) completely describe the evolution of the physical quantities of the EBC in the reaction with high-temperature water vapour. The equations mentioned above are expressed as follows:

(43) $$\varepsilon_{z}=\frac{\partial \mu_{z}}{\partial t}=\frac{1}{\tilde{\lambda }+2G}\left[\frac{\mu^{0}}{\Omega }\left(\hat{\mu }-1\right)+L\bar{l}\hat{l}\right]\;\sigma_{x}=\sigma_{y}=\frac{-2G}{\tilde{\lambda }+2G}\left[\frac{\mu^{0}}{\Omega }\left(\hat{\mu }-1\right)+L\bar{l}\hat{l}\right]$$

(44) $$\frac{\partial \hat{\mu }}{\partial t}=\frac{D^{**}}{h^{2}}\frac{\partial^{2}\hat{\mu }}{\partial {\hat{z}}^{2}}+\frac{L^{*}\bar{l}}{\mu_{0}}\frac{\partial \hat{l}}{\partial t}$$

(45) $$\frac{\partial \hat{l}}{\partial t}=\left[\frac{{k\mu }^{0}\left(\hat{\mu }-1\right)}{{\bar{l}\Omega }^{2}\left(\tilde{\lambda }+2G\right)}+\left[\frac{\mathrm{kL}}{\Omega \left(\tilde{\lambda }+2G\right)}+\left(\vartheta -\frac{3\beta }{\Omega }\right)k\right]\hat{l}\right]H\left(1-\hat{l}\right)\;=\left[\frac{{k\mu }^{0}}{{\bar{l}\Omega }^{2}}\left(\hat{\mu }-1\right)+k^{**}\hat{l}\right]H\left(1-\hat{l}\right)$$

where $\hat{z}=z/h\in \left[0,1\right]$, $\hat{\mu }=\mu /\mu^{0}\in \left[0,1\right]$ and $\hat{l}=l/\bar{l}\in \left[0,1\right]$.

To solve the aforementioned partial differential equations, the constraints of the boundary conditions are also required. For the corrosion process of the EBC under high-temperature water vapour, the potential chemical of the gas environment outside the rare-earth silicate coating and at the outer surface was assumed to be zero. At the interface between the EBC and the substrate, there was no water vapour diffusion from the bottom to the top, and the chemical potential gradient was always zero. The rare-earth silicate coating did not undergo selective corrosion with high-temperature water vapour initially, and the degree of the chemical reaction was zero in the entire space. In addition, the initial overall normalised chemical potential of the coating was 1, and the actual chemical potential was negative, which reflected the direction of the chemical reaction. The mathematical description of the boundary conditions is as follows:

$$\hat{\mu }\left(1,t\right)=0,\frac{\partial \hat{\mu }}{\partial \hat{z}}{|}_{\hat{z}=0}=0$$

$$\hat{l}\left(\hat{z},0\right)=0,\hat{\mu }\left(\hat{z},0\right)=1$$

(46) $$\hat{l}\left(\hat{z},0\right)=0,\hat{\mu }\left(\hat{z},0\right)=1$$

Based on the provided boundary conditions, the chemical potential of the EBC system under high-temperature water vapour conditions and the evolution of the chemical reaction degree over time can be solved. Subsequently, the result can be substituted into Equation (42) to obtain the overall stress and strain distribution and evolution mechanisms of EBC.

## 4. Results and Discussion

### 4.1. Methods of Solving Differential Equations

The partial differential equation (PDE) module in the commercial software COMSOL solves the abovementioned control equations. The COMSOL PDE module is based on the Newton iteration method and provides the general form of the differential equation solving function represented by Equation (47):

(47) $$e_{a}\frac{\partial^{2}u}{\partial t^{2}}+d_{a}\frac{\partial u}{\partial t}+\nabla \cdot \left(-c\nabla u-\alpha u+\gamma \right)+\beta \cdot \nabla u+\mathrm{au}=f$$

where u indicates the variable to be solved; $e_{a}$, $d_{a}$, and c are the mass, damping and diffusion coefficients, respectively. Moreover, α, β and γ represent the conserved flux convection coefficient, convection coefficient, and conserved flux source, respectively. Further, a represents the boundary absorption coefficient, and f is the source term.

The physical quantities of the EBC system were constant in the in-plane direction. They evolved in the direction perpendicular to the in-plane, which is considered a one-dimensional model. The corresponding boundary conditions were set in the model. In addition, the normalised dimensionless length was used for the calculation. The length of the one-dimensional linear model was 1, which represents the entire EBC thickness. The overall number of grids was 200.

### 4.2. Dimensionless Time Characteristic Parameters

The existing research related to corrosion in EBC systems underwater vapour is limited. Therefore, it was difficult to obtain specific values for most thermodynamic and material parameters in the model. Thus, the macro assignment method was adopted in the calculation of the differential equations in the model, and the specific values were ignored. The calculation results in only reflect qualitative laws, which can be used as a reference.

The degree of corrosion reaction and the corresponding mass coefficient in the chemical potential evolution equations reflect the diffusion behaviour of water vapour and the speed of the corrosion chemical reaction, respectively. Equations (44) and (45) are expressed as follows:

(48) $$\frac{\partial \hat{\mu }}{\partial t}=\frac{1}{\tau_{D}}\cdot {\left(1-\hat{l}\;*0.27\right)}^{-3/2}\cdot \frac{\partial^{2}u}{\partial z^{2}}+B\frac{\partial \hat{l}}{\partial t},{\;\tau }_{D}=\frac{h^{2}}{D^{**}}$$

$$\frac{\partial \hat{l}}{\partial t}=\left[\frac{k^{*}\mu^{0}}{\bar{l}\Omega }\cdot \left(\hat{\mu }-1\right)+\frac{1}{\tau_{R}}\cdot \hat{l}\right]\cdot H\left(1-\hat{l}\right),{\;\tau }_{R}=k^{**}\;$$

(49) $$\frac{\partial \hat{l}}{\partial t}=\left[\frac{k^{*}\mu^{0}}{\bar{l}\Omega }\cdot \left(\hat{\mu }-1\right)+\frac{1}{\tau_{R}}\cdot \hat{l}\right]\cdot H\left(1-\hat{l}\right),{\;\tau }_{R}=k^{**}\;$$

where $\tau_{D}$ and $\tau_{R}$ represent the characteristic time scale of water vapour diffusion and corrosion chemical reaction, respectively. The dimensionless parameter, λτ=τDτR, represents the ratio of two-time scales.

The influence of different dimensionless parameters, $\lambda_{\tau },$ on the two was analysed and discussed by comparing the evolution process of the normalised chemical potential and normalised chemical reaction degree. Except for $\lambda_{\tau }$, the other parameters used are listed in Table 1.

### 4.3. Evolution of Chemical Reaction

**Figure 2:**
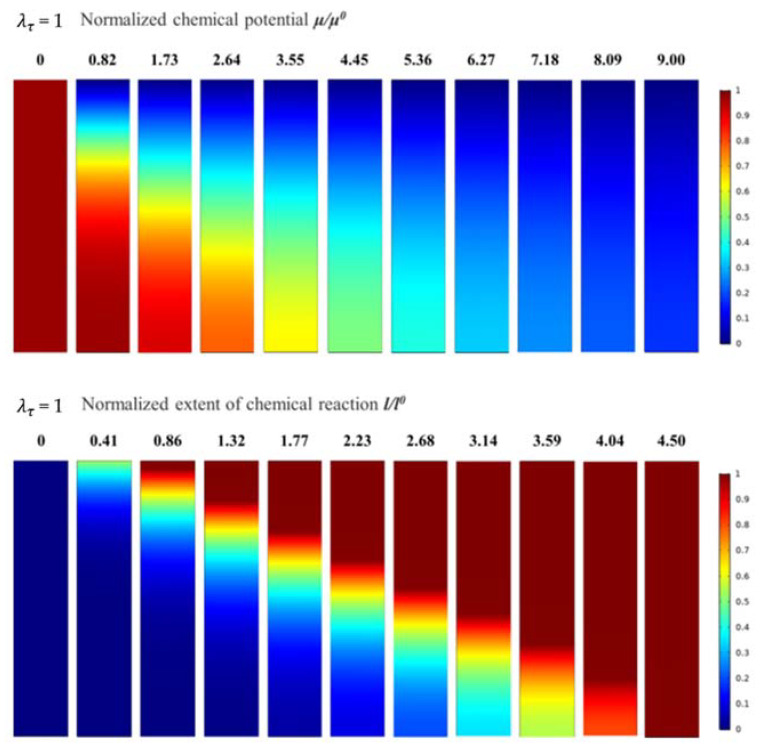
The evolution process of normalised chemical potential and normalised chemical reaction degree when $\lambda_{\tau }$ = 1.

The calculation results of the normalised chemical potential and normalised chemical reaction degree were used to analyse and discuss the corrosion mechanisms of water vapour in the EBC system. For $\lambda_{\tau }$ = 1, the evolution of the normalised chemical potential and the normalised chemical reaction degree are shown in Figure 2. The plane in Figure 2 corresponds the xz plane in Figure 1. The number above the figure indicates the time elapsed during the corrosion process. All descriptions of time are qualitative and only represent the relative length of time. In the evolution process of the normalised chemical potential, the red area represents the area with high chemical potential, indicating that the water vapour content is low, and the blue area represents the area with low chemical potential, which shows that this area already contained diffused water vapour. In addition, as the corrosion process developed, the normalised chemical potential gradually decreased from 1 to 0, indicating that water vapour gradually diffused from the outside to the inside of the EBC system. When the normalised chemical potential of all areas decreased to 0, the system reached a steady state, and the water vapour did not continue to diffuse.

In the evolution process of the normalised chemical reaction degree, the red area represents the area with more chemical reactions, the top corrosion area. The blue area represents the area with a lower chemical reaction degree, which indicates that the area had not been corroded by water vapour. It can be seen that as the water vapour diffused into the EBC system, corrosion developed, and the corroded area gradually expanded to the inside and finally reached a steady state. It should be noted that the time required for the water vapour to reach a steady state was slightly longer than that of the corrosion reaction.

**Figure 3:**
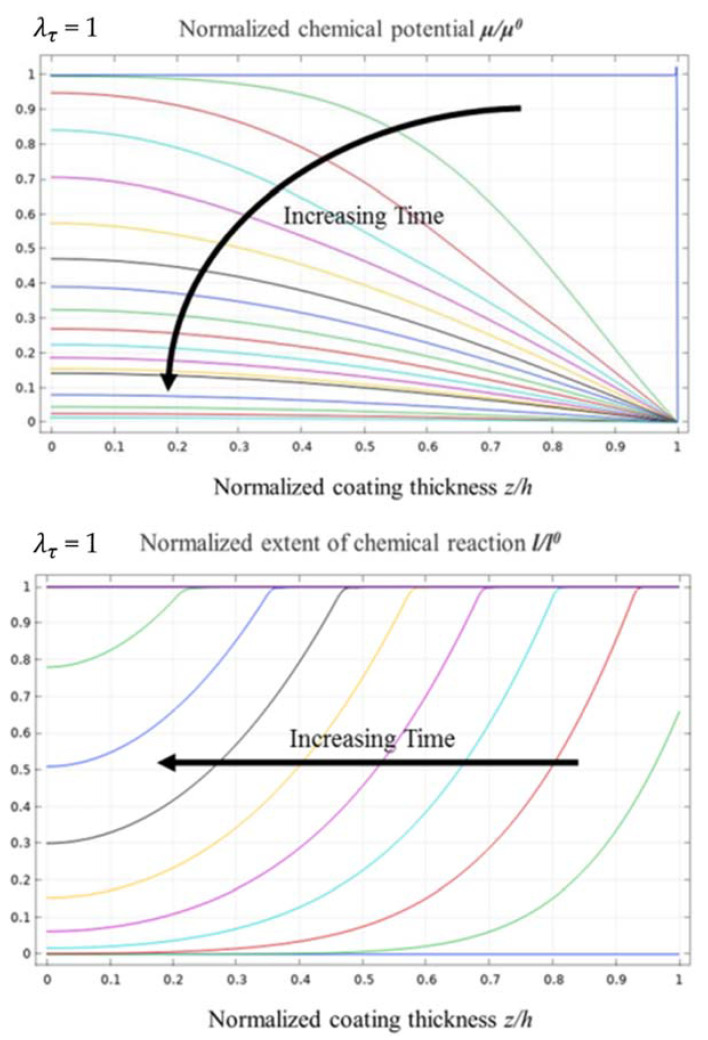
Evolution curves of normalised chemical potential and normalised chemical reaction degree when $\lambda_{\tau }$ = 1.

Figure 3 shows the evolution curves for the normalised chemical potential and the normalised chemical reaction degree when $\lambda_{\tau }$ = 1. The vertical axes of the figure represent the normalised chemical potential and the normalised chemical reaction degree, respectively. From the evolution curve of the normalised chemical potential, it can be observed that water vapour diffused faster into the EBC system in the early stage of diffusion. As the time increased, the depth of diffusion increased, and the water vapour diffusion speed decreased significantly due to a relatively large chemical potential gradient between the EBC system and the external environment. When the water vapour started to diffuse into the bottom of the EBC system, the potential chemical gradient gradually decreased, which resulted in a slower diffusion rate of water vapour. 

According to the evolution curve of the normalised chemical reaction degree, the variation in the gradient of the chemical reaction degree between the un-corroded and corroded areas is relatively low, and the curve is smooth. This indicates a certain thickness of the transition area between the un-corroded and corroded areas, and the transition area accounts for approximately 80% of the overall thickness of the EBC system. Furthermore, the front of the corrosion area at the top of the EBC system is not evident.

**Figure 4:**
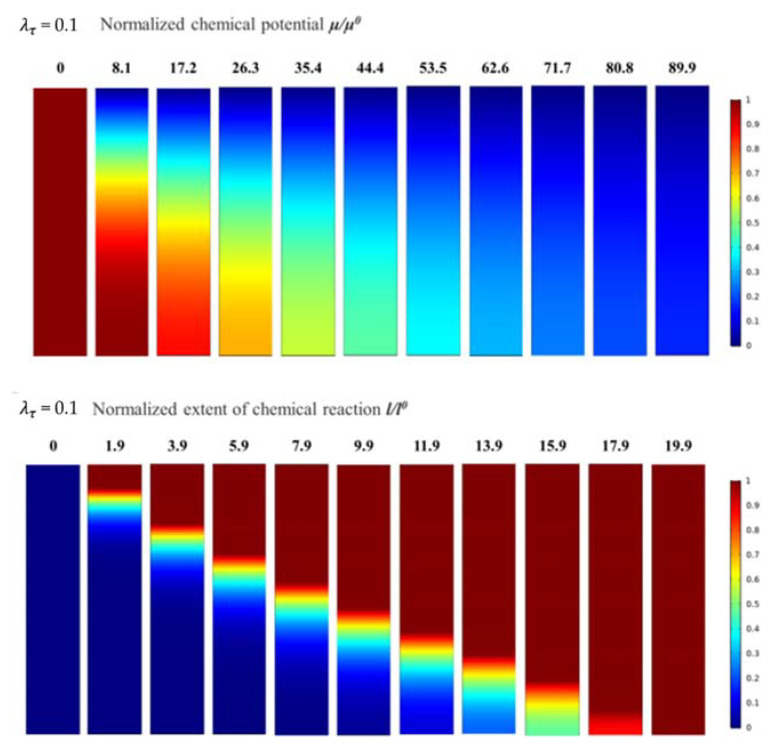
The evolution process of normalised chemical potential and normalised chemical reaction degree when $\lambda_{\tau }$ = 0.1.

When $\lambda_{\tau }$ = 0.1, the evolution of the normalised chemical potential and normalised chemical reaction degree is as shown in Figure 4, and the process of evolution is quite similar to that when $\lambda_{\tau }$ = 1. However, the boundary between the corroded and un-corroded areas is more evident. In addition, the main difference is reflected in the time scales for the normalised chemical potential and the normalised chemical reaction degree reaching a steady state. Due to the decrease in $\lambda_{\tau }$, the characteristic time scale of diffusion was longer than that of the chemical reaction. The diffusion process was significantly slower than the chemical reaction, which ultimately slowed down the progress of the chemical reaction.

**Figure 5:**
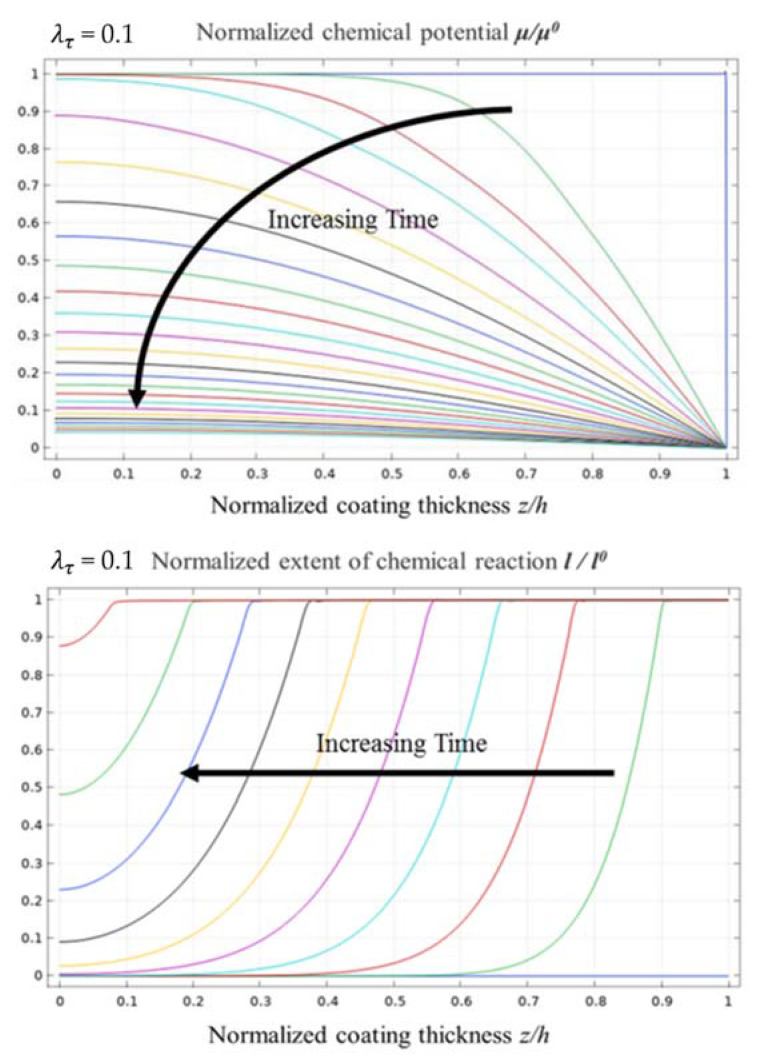
Evolution curves of normalised chemical potential and normalised chemical reaction degree when $\lambda_{\tau }$ = 0.1.

Figure 5 shows the evolution curves of the normalised chemical potential and the normalised chemical reaction degree when $\lambda_{\tau }$ = 0.1, which are considerably similar to those when $\lambda_{\tau }$ equals to 1. Therefore, the change in $\lambda_{\tau }$ had a weak effect on the distribution of water vapour in the diffusion process and only affected the time required for the diffusion of water vapour to reach a steady-state distribution.

Further, the gradient of the chemical reaction between the un-corroded and corroded areas is higher, and the curves are steeper than those when $\lambda_{\tau }$ = 1. The corrosion transition zone in the coating is approximately 30% of the overall thickness of the EBC system. The front of the corrosion zone at the top of the EBC system is still not evident.

**Figure 6:**
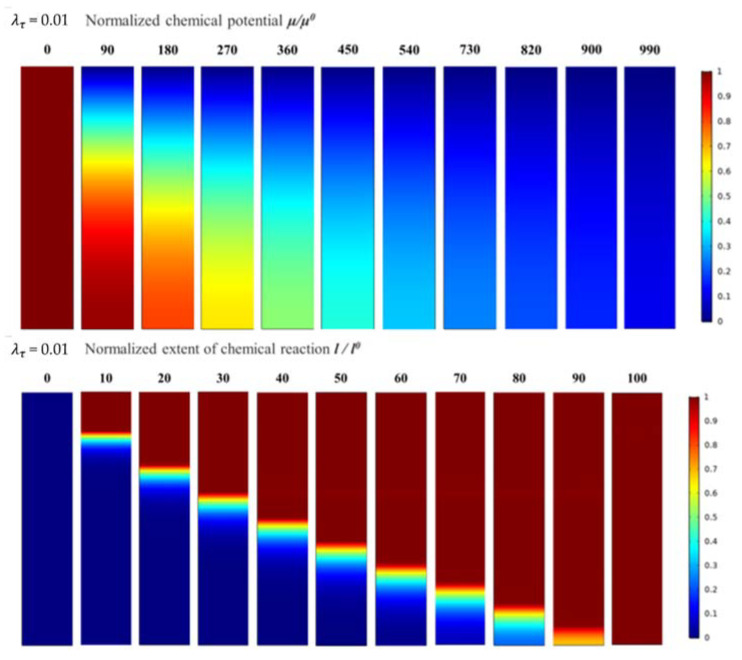
The evolution process for normalised chemical potential and normalised chemical reaction degree when $\lambda_{\tau }$ = 0.01.

When $\lambda_{\tau }$ equals 0.01, the evolution of the normalised chemical potential and normalised chemical reaction degree is as shown in Figure 6. The evolution process of the normalised chemical potential and normalised chemical reaction degree is consistent with the previous results. Subsequently, the time scales for the normalised chemical potential and normalised chemical reaction degree reaching a steady state increased significantly.

It is worth noting that the boundary between the corroded and un-corroded areas was further narrowed, and the transition area between the two was no longer evident. The front edge of the top corrosion area is clear, which is closer to the actual water vapour corrosion morphology of the rare-earth silicate EBC system.

**Figure 7:**
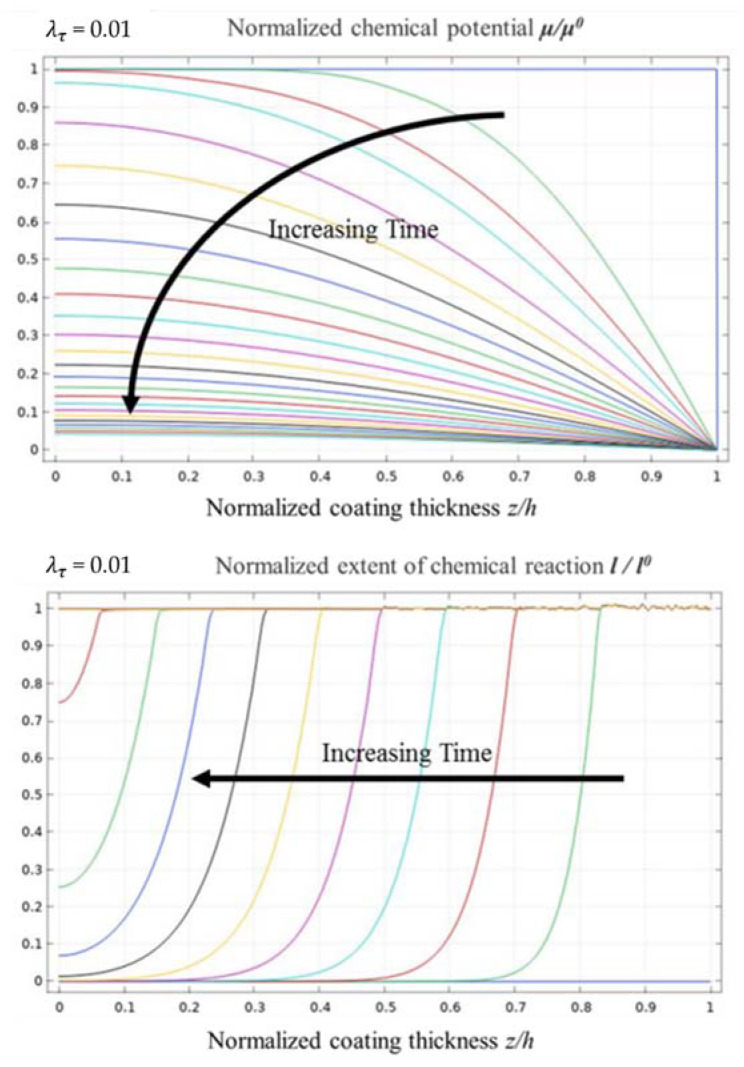
Evolution curves of normalised chemical potential and normalised chemical reaction degree when $\lambda_{\tau }$ = 0.01.

Figure 7 shows the evolution curves of the normalised chemical potential and normalised chemical reaction degree when $\lambda_{\tau }$ equals 0.01. Compared with the evolution curves of the normalised chemical potential when $\lambda_{\tau }$ equals 1 and 0.1, the evolution process is still quite similar. From the evolution curves of the normalised chemical reaction degree, the water vapour corrosion rate of the EBC system remained the same in the second half of the time span. In the initial period of the corrosion process, the corrosion rate was relatively fast, but the effect was not evident. Further, the change in the degree of the chemical reaction between the un-corroded and corroded area was relatively high; the front of the corrosion reaction was significantly evident, and the transition area was approximately 10% of the entire EBC system.

## 5. Conclusions

In this study, based on the thermodynamics of the continuum and the actual water vapour corrosion mechanisms of the EBC system, a theoretical model of high-temperature water vapour corrosion coupled with deformation, mass diffusion and the chemical reaction was established. First, the basic equilibrium equations of force, mass and energy were established. Then, based on the Helmholtz free energy dissipation inequality and chemical dynamics, the coupled constitutive equations of multi-field interaction were established, and the evolution equations of key physical quantities were derived. To consider the essential difference between mass diffusion and chemical reaction in the free energy and dissipation of a highly coupled system, the concentration of the diffusion substance and the degree of the chemical reaction were regarded as independent state variables. As the corrosion of the EBC system mainly occurs under long-term high-temperature conditions, the temperature change was ignored, and a special isothermal model was established to reflect the actual situation accurately. To better describe the actual corrosion situation, the concentration term in the final evolution equation was replaced with the chemical potential of water vapour.

The theoretical model was found suitable for solving the stress and strain fields, water vapour concentration distribution and coating corrosion degree distribution in any stage of the EBC system from the intact initial state to the water vapour corrosion process. The distribution of the water vapour concentration and the distribution of the corrosion degree was similar. Water vapour diffused from the outer surface of the coating into the EBC system and selectively corroded. The corrosion area of water vapour gradually penetrated the interior of the EBC system. In this process, the thickness change of the corrosion area depended on the diffusion rate of water vapour, which had the greatest impact on the corrosion process of the EBC. This is because the rate of water vapour diffusion was much slower than the reaction rate of the rare-earth silicate with water vapour. If the diffusion rate of water vapour is insufficient, the reaction of rare-earth silicate is significantly hindered. Therefore, the front of the top corrosion zone of the rare-earth silicate EBC system was quite evident, and a clear dividing line existed between the un-corroded and corroded areas.

With the development of the corrosion process, the diffusion speed for water vapour gradually slowed down owing to the increase in the diffusion distance. When the water vapour diffused to the entire area, the diffusion rate further slowed down because of the reduced chemical potential difference. Therefore, the corrosion area exhibited a parabolic dynamic evolution trend.

## Figures and Tables

**Table 1:** Main thermodynamic parameters used in the model.

Parameter	Value
Volume of water molecule, Ω/m^−3^	10^−28^
Initial chemical potential, μ^0^	−0.5 × 10^−20^
Chemical expansion coefficient, β	−0.6 × 10^−5^
Reaction-chemical potential coupling coefficient, N	2 × 10^−24^
Chemical potential constant, ς	50 × 10^−49^
Characteristic time scale of water vapour diffusion, τ_D_	2000
Characteristic time scale of corrosion reaction, τ_R_	20

## Data Availability

Data is contained within the article.
